# Optimization of carotenoid extraction of a halophilic microalgae

**DOI:** 10.1371/journal.pone.0270650

**Published:** 2022-08-02

**Authors:** Shanling Gan, Shengjia Liang, Qiman Zou, Changhua Shang

**Affiliations:** 1 Key Laboratory of Ecology of Rare and Endangered Species and Environmental Protection, Ministry of Education, College of Life Sciences, Guangxi Normal University, Guilin, China; 2 Guangxi Key Laboratory of Landscape Resources Conservation and Sustainable Utilization in Lijiang River Basin, Guangxi Normal University, Guilin, China; 3 School of Life Sciences, Sun Yat-Sen University, Guangzhou, China; Zhengzhou University, CHINA

## Abstract

*Dunaliella parva* can produce abundant carotenoids under certain conditions. This paper optimized the extraction efficiency of carotenoids from *D*. *parva*. Different organic solvents were examined to determine the most suitable solvent for the extraction. After the determination of the solvent (dimethylsulfoxide, DMSO), the extraction conditions including time, temperature, and volume were then optimized to maximize the extraction efficiency of carotenoids from *D*. *parva* using response surface methodology. DMSO was identified as the most suitable solvent. The optimal extraction conditions were as follows: temperature of 57.2°C, time of 11.35 min, the volume of 410 μl, and the optimal extraction efficiency reached 0.517‰. The results showed that the optimal extraction efficiency (0.517‰) improved 31.69% in comparison to the initial extraction efficiency (0.3926‰). In addition, The optimal levels of three influence factors (temperature of 57.2°C, time of 11.35 min, volume of 410 μl) decreased compared with the initial levels (temperature of 60°C, time of 20 min, volume of 1000 μl). In this paper, Central Composite Design (CCD) was used to optimize the extraction efficiency of carotenoids from *D*. *parva*, which would lay the groundwork for the extraction and utilization of carotenoids from *D*. *parva* in the future.

## 1. Introduction

Carotenoids are important natural pigments and the main source of vitamin A in the body. At the same time, they also have the following functions such as antioxidant, immune regulation, anti-cancer, and anti-aging effects, which have been used for human benefits and many industrial applications [1–3]. Therefore, carotenoids have a very high economic value.

Carotenoids are naturally present in organisms ranging from prokaryotes (e. g. bacteria) to eukaryotes (e. g. algae and higher plants) [4]. Natural carotenoids have received increasing attention in recent years because of their health benefits compared to synthetic carotenoids [5]. Natural carotenoids are produced by plants and algae, while synthetic carotenoids mainly are by-products of the distillation of coal [6]. Animals and people can not synthesize carotenoids by themselves, and just can rely on diet to absorb carotenoids and further metabolize them in the body to meet their own needs. As a result, a growing number of industries are synthesizing carotenoids in large quantities through artificial chemistry in order to meet the increasing demand in nutraceuticals, cosmetics, and pharmaceutical industries. However, the biggest disadvantage of synthetic carotenoids is that they can not be guaranteed to achieve the same effect as natural carotenoids in terms of the composition and efficacy. Synthetic carotenoids have an all-trans structure and do not inhibit some malignant tumor cells, whereas the 9-cis isomer of natural carotenoids can act as an anti-cancer agent [7]. Although carotene has long been produced by industrial synthesis, biosynthetic natural carotene is generally welcomed by people under the influence of the general trend of returning to nature. However, the content of carotene in most plants is very low, even in carrots is only 4 parts per 10,000.

Through painstaking efforts, scientists have found that some monocellular green algae such as *Dunaliella* can grow in high salt environments, and contain high levels of carotene [8]. Microalgae are considered as the richest sources of natural carotenoids, especially species of Chlorophyta such as *Dunaliella*, *Haematococcus pluvialis*, and various *Chlorella* [9]. Among them, *Dunaliella* is famous for its rich lutein, zeaxanthin and β-carotene [10]. *Dunaliella* is unicellular green algae, and is well known as the only biological source which can accumulate natural β-carotene (approximately 10%-15%) [11]. *Dunaliella* is an internationally recognized natural source of β-carotene. *Dunaliella* is halophilic single-cell microalgae accumulating large amounts of carotenoids (especially β-carotene) under stress conditions such as high temperature, high light, and high salt concentration [4, 12].

Response surface methodology (RSM) is a combination of mathematical and statistical techniques based on the fit of a polynomial equation to the experimental data [13]. RSM model includes Box–Behnken Design and Central Composite Design (CCD) [14]. RSM is an appropriate technique to optimize carotenoid extraction and maximize the antioxidant capacity of extracts from various plant resources [15]. But so far, there are few reports about the optimization of extraction conditions of carotenoids by RSM.

Strengthening the research and development of carotenoids in *Dunaliella* can not only expand the source of natural carotenoids, and meet the increasing demand of various fields, but also realize the full utilization of algae, and bring the considerable economic benefits. At present, researchers have carried out relevant studies on the optimization of the extraction process of carotenoids from *Dunaliella* and reported many methods for carotenoid extraction. The commonly used methods for carotenoid extraction include organic solvent extraction [16], saponification [17], supercritical carbon dioxide extraction [18, 19], and etc. But up to now, none of them can truly achieve economic, environmentally-friendly, and efficient extraction. Compared with other methods, organic solvent extraction is easy to operate and control the influencing factors, requires fewer equipment and lower cost, and has higher safety of whole process [9]. Therefore, various influencing factors of organic solvent extraction can be optimized to effectively promote the development and utilization of *D*. *parva*, and the commercial production of carotenoids.

The extraction of carotenoids with organic solvents gives good extraction rates without the use of complex apparatus. During the extraction process, the characteristics of the algae (water content, cell wall, and carotenoids composition) and the extraction parameters (solvent, extraction time, and temperature) are the most important factors affecting the effective extraction of carotenoids. The choice of solvent is the most critical factor for the effective extraction of carotenoids, and the physical and chemical properties of extraction solvent and carotenoids need to be similar. Meantime, the type of extraction solvent can affect the optimal extraction temperature. When the extraction temperature is close to or above the boiling point of organic solvent, it can make the organic solvent volatilize, which affects the extraction amount. Therefore, the influence of temperature can be very major. In addition, longer extraction time favors higher extraction yields, but the combination of different temperatures and times can lead to the generation of side reactions. Overall, it is essential to optimize the extraction of carotenoids from a halophilic microalgae *D*. *parva* using central composite design.

This paper selected *D*. *parva* as raw material, choose three influence factors with five levels for response surface analysis and evaluated the interaction among various factors and their effects on carotenoid extraction in *D*. *parva*. In addition, this paper established the relationship between extraction efficiency and influence factors through regression analysis to optimize the conditions of carotenoid extraction. These results will provide a good basis for the extraction and utilization of carotenoids in *D*. *parva*.

## 2. Materials and methods

### 2.1. Algae species and growth condition

*D*. *parva* was purchased from Freshwater Algae Culture Collection at the Institute of Hydrobiology. *D*. *parva* was cultured in Dunaliella medium under a light intensity of 34 μmol/m^2^/s illumination at 26°C with 14 h light/10 h dark cycle. The algae bottles gently swirled once or three times each day by hand.

### 2.2. Extraction of carotenoid and the single factor test

The single factor test was performed to compare the extraction efficiency of different solvents such as petroleum ether, ethyl acetate, 95% ethanol, ethanol, DMSO, and acetone according to the solubility of carotenoid. 1 ml of algal culture was centrifuged at 15,294 g for 5 min, and the supernatant was discarded. Then 1 ml of each of the six solvents was added to the algal cell pellet and well mixed. After mixing, the mixture was treated at 60°C for 20 min and then centrifuged at 12000 rpm for 2 min. Finally, the OD values of supernatant (300 μl) were measured using Epoch 2 Microplate Spectrophotometer at 480 nm, 649nm, and 665nm to study the extraction effects of different solvents. The cell pellet of 1 ml of algal culture was dried for 12 h at 60°C to determine the dry weight of alga. Firstly, the empty 1.5 ml centrifuge tube was weighed. Secondly, 1 ml of algal culture was added to the empty 1.5 ml centrifuge tube, and the supernatant was discarded after centrifugation at 15,294 g for 2 min. At last, the 1.5 ml centrifuge tube containing the fresh algal cell pellet was dried at 60°C for 12 h and weighed again. The dry cell weight of *D*. *parva* was determined routinely by measuring the weight difference of dried 1.5 ml centrifuge tube after drying the algal cells. Three replicates were performed for each group.

### 2.3. Quantitative determination of extraction efficiency

The extraction efficiency of carotenoid was calculated according to the following formula after obtaining the OD values of samples at 480 nm, 649nm, and 665nm.


$$chla=12.47\times A_{665}-3.62\times A_{649}$$

$$chlb=25.06\times A_{649}-6.5\times A_{665}$$

$$C=(1000\times A_{480}-1.29\times Chla-53.78\times Chlb)/220$$

$$Y=\frac{C\times V}{m\times 1000\times 1000}\times 1000‰$$

In this formula, *Chla* is the concentration of chlorophyll a (μg/mL); *chlb* is the concentration of chlorophyll b (μg/mL); C is the content of carotenoids in the sample (μg/mL); Y is the extraction efficiency of carotenoids (‰); V is the volume of DMSO (μL); M is the dry weight of the sample (mg).

### 2.4. Experimental design

In this study, *D*. *parva* was used as raw material. After the optimal solvent was determined, the extraction efficiency was used as response value (Y), and extraction time (A), extraction temperature (B), and the volume of extraction solvent (DMSO, C) were used as influence factors. Central Composite Design (CCD) of response surface methodology (RSM) was used to carry out an experimental design of three factors with five levels (shown in Table 1). The design consisted of 20 random tests (shown in Table 2). All tests were performed in triplicate and the mean of the extraction efficiency of carotenoid was calculated. Extraction conditions of carotenoids were investigated and optimized through model-fitting analysis, residual analysis, variance analysis, and 3D surface analysis of interaction terms. Response surface analysis was performed using Design-Expert 10.0.4 software.

**Table 1:** The levels of the variables in this study.

Factors	Levels				
	-α	-1	0	+1	+α
A (time, min)	6.59	10	15	20	23.41
B (temperature,°C)	33.18	40	50	60	66.82
C (volume, μL)	242.96	300	550	1000	1067.04

**Table 2:** The design of 20 random tests.

Group	Temperature (°C)	Time (min)	volume (μL)
6	40	20	900
16	50	15	655
5	40	10	900
3	60	10	410
10	50	23.41	655
1	40	10	410
13	50	15	242.96
17	50	15	655
9	50	6.59	655
18	50	15	655
12	66.82	15	655
8	60	20	900
19	50	15	655
7	60	10	900
4	60	20	410
11	33.18	15	655
14	50	15	1067.04
20	50	15	655
15	50	15	655
2	40	20	410

## 3. Results and discussion

### 3.1. Effects of different solvents on extraction efficiency of carotenoid

**Fig 1:**
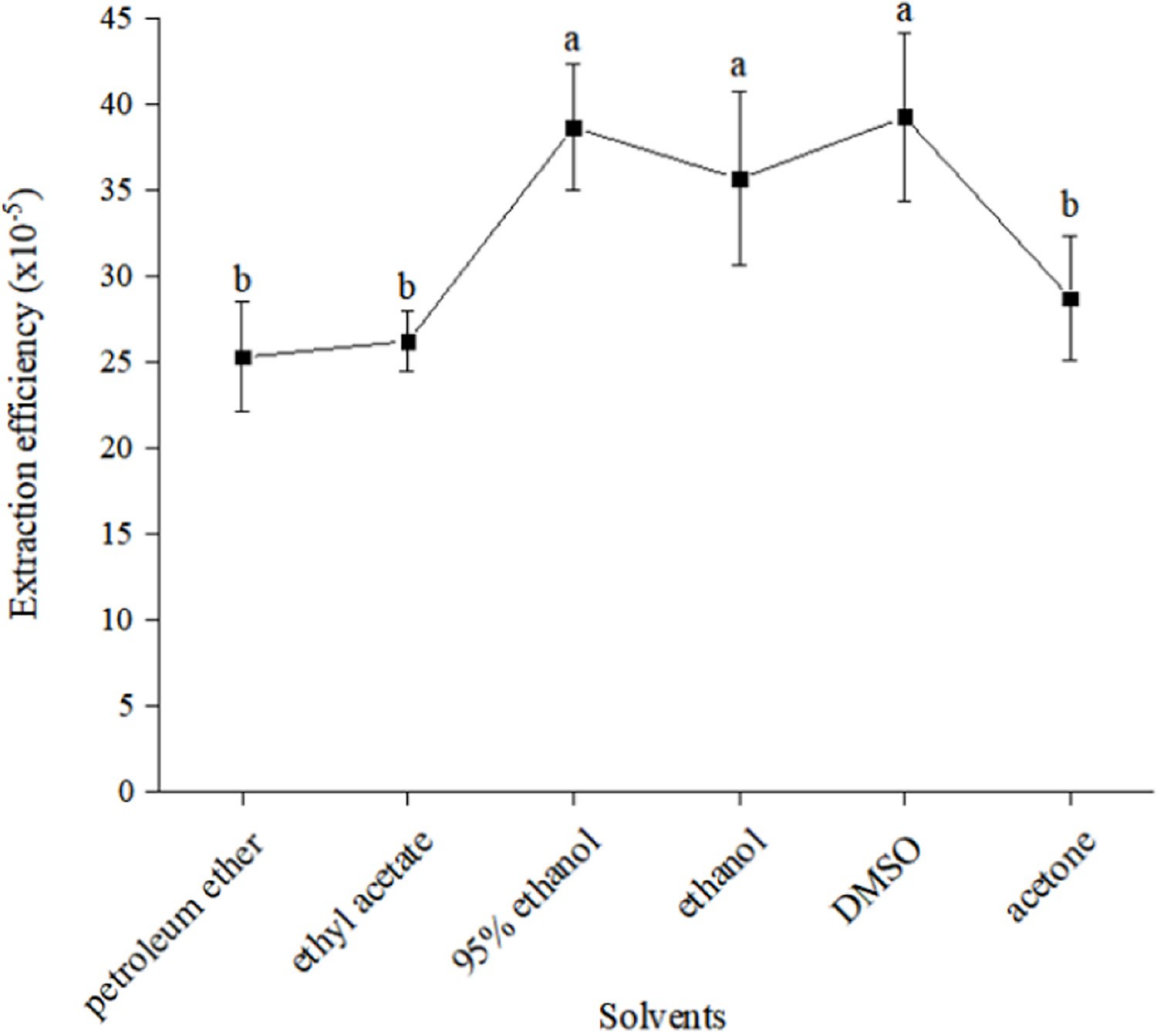
Effects of different solvents on extraction efficiency of carotenoids. Two items with same letter have no significant difference. Two items with different letter have significant difference.

The effects of different solvents on the extraction efficiency of carotenoids were shown in Fig 1. Carotenoids can be dissolved in most organic solvents, but different solvents have different extraction efficiency. Among six solvents in this experiment, DMSO, 95% ethanol and ethanol had the higher extraction efficiency. This may be due to the strong cell permeability of DMSO compared to other solvents. Therefore, DMSO was chosen as the extraction solvent for optimization of extraction conditions in the subsequent experiments.

### 3.2. Regression model and statistical test

The results of extraction efficiencies of carotenoid under different extraction conditions were calculated based on the design of CCD and shown in Table 3. Using design expert to perform regression analysis and curve fitting of the data (Table 3), the relationship among extraction efficiency (Y) and response variables such as extraction time (A), temperature (B), and extraction volume (C) was obtained, which was expressed by the following equation.


$$Y=54.09-1.12A+4.43B-0.74C+5.32AB+4.68AC-2.71BC-1.57A^{2}-5.54B^{2}-1.86C^{2}+3.94ABC-3.34A^{2}B+0.53A^{2}C-3.43AB^{2}$$

**Table 3:** Results of extraction efficiency of carotenoid.

Group	A (Time, min)	B (Temperature,°C)	C (Volume, μL)	Extraction efficiency (X10^-5^)	
				Experimental value	Predicted value
1	10	40	410	51.4	52.1
2	20	40	410	30.2	31.0
3	10	60	410	56.2	57.0
4	20	60	410	40.6	41.3
5	10	40	900	54.9	55.7
6	20	40	900	36.7	37.4
7	10	60	900	33.2	33.9
8	20	60	900	52.0	52.7
9	6.59	50	655	52.6	51.5
10	23.41	50	655	48.8	47.8
11	15	33.18	655	32.0	31.0
12	15	66.82	655	46.9	45.9
13	15	50	242.96	51.1	50.1
14	15	50	1067.04	48.6	47.6
15	15	50	655	52.3	54.1
16	15	50	655	55.7	54.1
17	15	50	655	54.8	54.0
18	15	50	655	50.8	54.1
19	15	50	655	55.7	54.1
20	15	50	655	54.8	54.1

**Fig 2:**
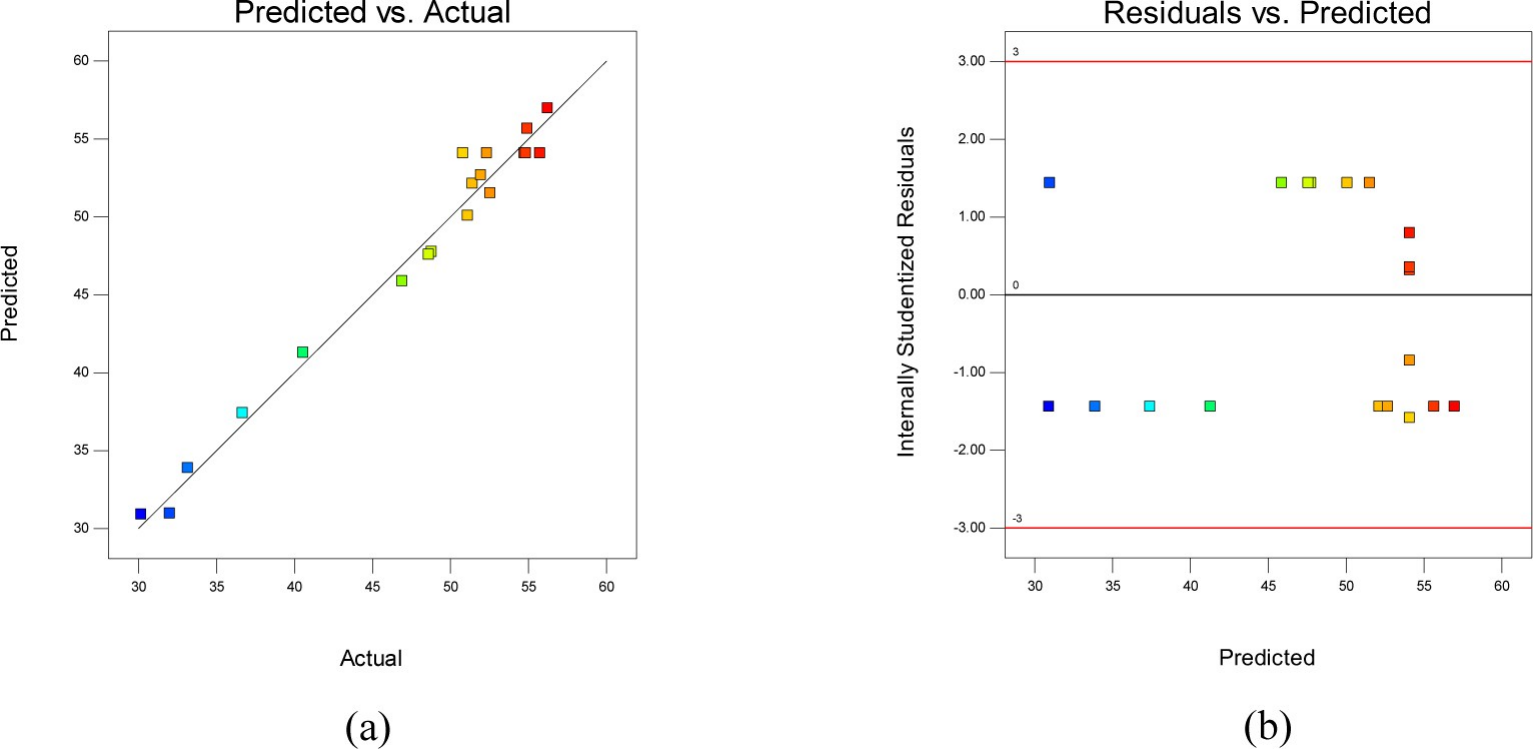
Statistical analysis of the model. (a) Comparison of actual and predicted response values. (b) Comparison of internally studentized residuals and predicted response values.

The predicted values were obtained using Design Expert to analyze the actual extraction efficiency in Table 3. The relationship between the predicted values and the actual response values (i. e. extraction efficiency) was shown in Fig 2A. As shown in Fig 2A, most of the points were distributed near a straight line, which indicated that there was a good correlation between the experimental values and the theoretical values. Fig 2B showed the relationship between predicted response values and residuals. If the distribution of the points is more scattered, it means that the model is more reliable. As shown in Fig 2B, most of the points were in irregular distribution, so the fitted model was reliable and could provide guidance for optimizing the extraction conditions.

In order to test whether the selected model was effective and whether the predicted results were reliable, residual and variance analysis was carried out for the regression equation established by the software (Design Expert). In addition, further confirm the influence of extraction time (A), temperature (B), and extraction volume (C) on the extraction efficiency of carotenoids.

According to Table 4, the F value of the model was 20.34 with *p*-value (0.0007<0.001), which indicated that the correlation between the dependent variable (extraction efficiency) and the independent variables (influence factors) in the regression model was highly significant, and response surface model could be used for subsequent optimization. The *p*-value was less than 0.01, which suggested that the model terms were very significant. Within the range of selected factors, P_B_, P_AB_, P_AC_, P_B_^2^, and P_ABC_ were all less than 0.01, which were extremely significant. The values of P_BC_, P_A_^2^, P_C_^2^, P_A_^2^_B_, and P_AB_^2^ (less than 0.05) were significant, while the significance of other items was not significant. F value of a single factor represents its influence degree on the response value. Therefore, in this experiment, the influence degree of each factor on the extraction efficiency of carotenoid was as follows: extraction temperature (B)> extraction time (A)> extraction volume (C). Extraction temperature (B) had the most significant effect. The coefficient R^2^ stood for the reliability of the model. The value of R^2^ (0.9778) indicated that the correlation between the regression model and the actual values was high and this model had a good potential for response prediction for the experimental design. The coefficient of variation CV (4.73%), reflecting the repeatability of the test, is significantly less than 10%, which indicated that the test has high reliability and precision. The precision (13.731) was significantly greater than 4, indicating a strong guiding effect on the experimental design. In summary, the regression model was well established.

**Table 4:** Analysis of variance for model.

Source	Sum of squares	d. f.	Mean square	F value	*p* value	Significance
Model	1360.84	13	104.68	20.34	0.0007	**
A	7.07	1	7.07	1.37	0.2856	
B	111.15	1	111.15	21.60	0.0035	**
C	3.12	1	3.12	0.61	0.4654	
AB	226.74	1	226.74	44.06	0.006	**
AC	175.31	1	175.31	34.07	0.0011	**
BC	58.92	1	58.92	11.45	0.0148	*
A^2^	35.75	1	35.75	6.95	0.0388	*
B^2^	442.32	1	442.32	85.95	<0.0001	**
C^2^	49.93	1	49.93	9.70	0.0207	*
ABC	123.95	1	123.95	24.09	0.0027	**
A^2^B	37.06	1	37.06	7.20	0.0364	*
A^2^C	0.95	1	0.95	0.18	0.6830	
AB^2^	38.95	1	38.95	7.57	0.0332	*
Residual	30.88	6	5.15			
Lack of fit	10.64	1	10.64	2.63	0.1657	
Pure error	20.23	5	4.05			
Sum	1391.72	19				
R^2^	0.9778					
Adj R^2^	0.9297					
CV%	4.73					
Precision	13.731					

Note

* (*p*<0.05) represented significant, and

** (*p*<0.01) represented extremely significant. d. f. represented degrees of freedom.

### 3.3. Interaction among influence factors

**Fig 3:**
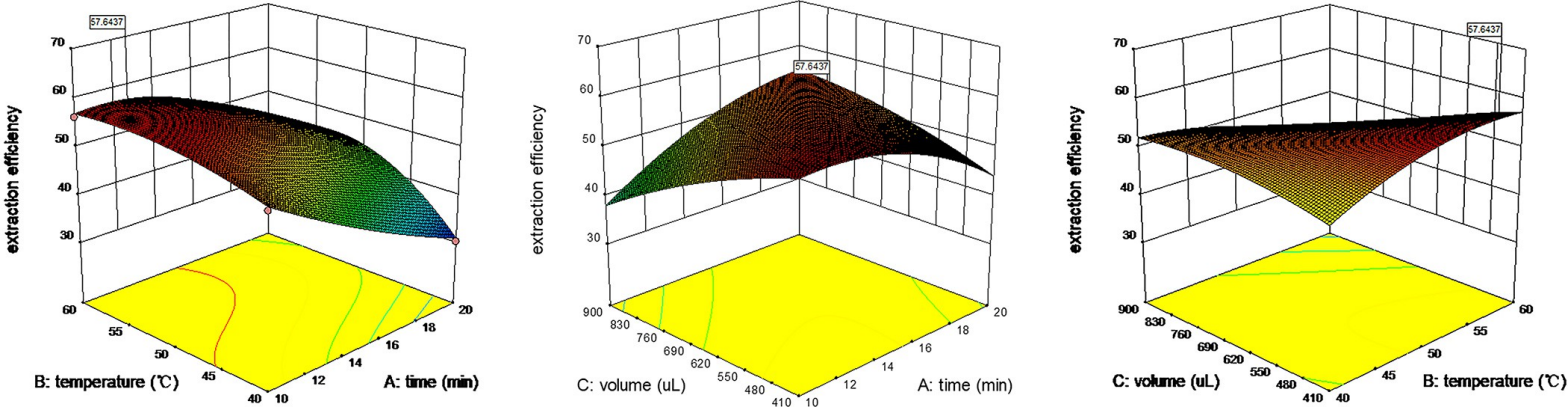
Response using CCD obtained by plotting: (a) time vs. temperature; (b) time vs. volume; (c) temperature vs. volume.

By analyzing the above experimental results, it could be seen that extraction time (A), extraction temperature (B), and extraction volume (C) did not independently affect the response value of extraction efficiency, and there were interactions among the factors. To visually reflect the interactions between influence factors, three-dimensional response surface diagrams were drawn with Design Expert, which were shown in Fig 3A–3C.

The curved surface in Fig 3A was wavy. When the temperature was high, extraction efficiency changed little with the increase of extraction time, while when the temperature was low, extraction efficiency decreased significantly with the increase of extraction time. Fig 3A indicated that the interaction between extraction time and the temperature was significant under the condition of constant extraction volume. The curve in Fig 3B had a steep slope. The extraction efficiency of carotenoids increased significantly with the increase of extraction temperature and the decrease of extraction volume. Fig 3B indicated that temperature and extraction volume had a great interaction effect on extraction efficiency. The curved surface in Fig 3C was relatively gentle, which indicated that temperature and extraction volume had little interaction under the condition of constant extraction time. The above analysis (Fig 3A–3C) indicated that the interaction among extraction temperature, extraction time, and extraction volume had a certain effect on the extraction efficiency of carotenoids.

### 3.4. Determination and validation of optimal extraction conditions

Based on the model analysis and the prediction of optimal extraction conditions, the optimal extraction time was 11.35 min, the temperature was 57.157°C, and the volume was 410 μL. Under the optimal conditions, the predicted value of extraction efficiency was 0.576‰. In order to verify the accuracy of the regression model of the response surface method, the optimal extraction efficiency under the optimal extraction conditions was subjected to verification. The actual extraction efficiency of carotenoids was 0.517‰. The actual value was very close to the predicted maximum. Based on the above experimental result, it was proved that the model was reliable.

### 3.5. Related research of microalgae

Microalgae have been considered as a promising raw materials for biodiesel production, but biodiesel production from microalgae has not yet completely achieved commercialization, which is due to the higher energy costs related to the whole process chain such as cultivation, harvesting, dewatering and extraction. Researchers considered that producing bioactive substances such as omega-3 oils, vitamins and pigments, could counterweigh the higher cost of biodiesel production from microalgae.

As oleaginous microalgae, green algae *Dunaliella* genus had a lot of advantages including easy cultivation, high stress resistance to salinity and light, higher growth rate and oil content [20–22]. The bottleneck of higher cost of biodiesel production from microalgae have led to intensive studies about production of microalgal bioactives, which includes vitamins and pigments with nutraceutical and pharmaceutical applications [23, 24]. *Dunaliella* genus (such as *Dunaliella* salina, *Dunaliella tertiolecta* and *D*. *parva*) is a halotolerant microalgae, which is rich in natural carotenoids [25, 26]. However, few studies were reported about carotenoid separation from *Dunaliella* genus, which limited the development and application of microalgae.

Traditional methods are based on organic solvent extraction [27–29], which include grinding [30], enzyme-assisted extraction [31, 32], and ultrasonic extraction [33]. The most important characteristic of organic solvent extraction is that it is efficient without the change in the properties of carotenoids, but conventional solvent extraction usually requires large amounts of solvent, long extraction time and tedious steps [34]. Nowadays, for the sake of human health and environmental protection, researchers have developed a method to extract carotenoids from microalgae using a green solvent (vegetable oils) instead of the traditional organic solvent [35]. In addition, many new extraction methods are available, such as pressurized liquid extraction [36–38], supercritical fluid extraction (SFE) [39, 40], in situ extraction [41–43], aqueous two-phase extraction [44]. The SFE method is consistent with the concept of "green chemistry", which produces carotenoids with high safety, but this method requires expensive equipment and its extraction rate of carotenoids is lower than that of the organic solvent method. In situ extraction method can effectively avoid the harvesting process and achieve simultaneous microalgae culture and carotenoids extraction, which thus can reduce energy consumption and production cost, but this method has some problems such as low extraction rate. In summary, although more extensive and in-depth studies have been made to extract carotenoids from microalgae, there is no the perfect method with all advantages.

Organic solvent extraction is the common method for carotenoids extraction from microalgae such as *D*. *parva* [20–22]. Therefore, firstly, the best solvent with highest extraction effect (DMSO, 0.3926‰) was determined. Then CCD method was used to optimize the extraction conditions (tempreture, time and volume). At last, the optimized conditions (extraction time of 11.35 min, temperature of 57.2°C, volume of 410 μL) and the actual maximum of extraction efficiency (0.517‰) was obtained. The results showed that the optimal levels of extraction cnoditions (temperature of 57.2°C, time of 11.35 min, volume of 410 μL) significantly decreased compared with the initial extraction cnoditions (temperature of 60°C, time of 20 min, volume of 1000 μL). Through CCD optimization, the improved extraction efficiency (0.517‰) increased by 31.69% compared with the initial extraction efficiency (0.3926‰) under the conditions of lower energy consumption and the volume of 41%. This paper would lay a good foundation for the application of *D*. *parva* in the future.

## 4. Conclusion

This paper showed that DMSO had the best extraction effect followed by 95% ethanol, and petroleum ether had the worst extraction effect. Under the optimal extraction conditions (extraction time of 11.35 min, temperature of 57.2°C, volume of 410 μL), the optimal extraction efficiency of carotenoids from *D*. *parva* was 0.517‰. The results showed that the optimal extraction efficiency (0.517‰) improved 31.69% in comparison to the initial extraction efficiency (0.3926‰). In addition, The optimal levels of three influence factors (temperature of 57.2°C, time of 11.35 min, volume of 410 μL) decreased compared with the initial levels (temperature of 60°C, time of 20 min, volume of 1000 μL). In this study, CCD was used to optimize extraction conditions of carotenoids from *D*. *parva*, which would lay a good foundation for the extraction and utilization of carotenoids from *D*. *parva* in the future.
